# Autophagy Associated Genes (ARGs) ‐Based Predictive Model AIDPS for Prostate Cancer

**DOI:** 10.1111/jcmm.70213

**Published:** 2025-01-09

**Authors:** Zhiyi Zhao, Yongjin Yang, Zhou Sun, LianMing Fan, Lingyun Liu

**Affiliations:** ^1^ Department of Andrology The First Hospital of Jilin University Changchun China; ^2^ Department of Urology China‐Japan Union Hospital of Jilin University Changchun China; ^3^ Department of Urology The Second Hospital of Lanzhou University Lanzhou China; ^4^ Department of Urology, Xiangyang Central Hospital Affiliated Hospital of Hubei University of Arts and Science Xiangyang China

**Keywords:** AIDPS, Autophagy, Mendelian Randomization, Prognosis, Prostate cancer

## Abstract

Prostate cancer (PCa) is one of the most common cancers in men worldwide. Autophagy‐related genes (ARGs) may play an important role in various biological processes of PCa. The aim of this study was to identify and evaluate autophagy‐related features to predict clinical outcomes in patients with PCa. Single‐cell sequencing data and RNA sequencing data was included from public GEO and TCGA databases. Cells were clustered and annotated by dimension reduction cluster analysis. Epithelial cells, T cells and fibroblasts were isolated to explore their heterogeneity. Autophagy‐related genes were obtained from the HADb database. Survival analysis was conducted by K–M curve, and prognostic risk model was established using 101 machine learning algorithms. In addition, we performed gene colocalisation analysis and Mendelian randomisation analysis. Univariate Cox analysis was used to screen out prognostic genes from DEGs and ARGs in each dataset. Risk model was generated by artificial intelligence‐derived prognostic signature (AIDPS), which showed better prognostic performance in every dataset than other published models for PCa. The disease‐free period (DFS) of patients in the high‐risk group was significantly worse than that in the low‐risk group (all *p* < 0.05). The best model is the Ridge (C‐index 0.726). We found significant differences in IC50 values of the Dactinomycin_1811, Dactolisib_1057, Luminespib_1559 and Paclitaxel_1080 between groups. In SNP sites rs2743987 and rs7768988, there was a significant correlation between prostate hyperplasia and prostate cancer. Our ARG‐based predictive model AIDPS is a reliable and effective tool for prognosis and treatment of prostate cancer.

## 1 Introduction

Prostate cancer (PCa) ranks as the second most prevalent malignancy among males, resulting in a significant number of fatalities [1]. In the United States, PCa ranks second among the leading cancer causes [2]. The clinical outcomes for advanced prostate cancer, specifically referred to as distant prostate cancer, exhibit superior results compared to prostate cancer at early stage in the majority of cases [3]. While advanced age, race, family history and smoking are acknowledged as risk factors for PCa [4], no modifiable risk factors have been definitively established. In order to alleviate the burden imposed by PCa, heightened emphasis should be placed on the identification and mitigation of its risk factors. With the growing recognition of the disease and the widespread utilisation of PSA screening, an increasing number of patients are being identified at an early stage [5]. However, despite these advancements, prostate adenocarcinoma (PRAD) continues to be the third leading cause of mortality among men and poses a significant burden on public health [6]. Currently, localised PCa is managed through various approaches such as watchful waiting, active surveillance, surgery or radiotherapy, while the treatment of advanced PCa remains a complex challenge for healthcare professionals [7]. Despite the emergence of numerous clinical and molecular studies on prostate cancer, the identification of diagnostic and prognostic biomarkers has not yet been accomplished.

Autophagy, a biological process, involves the translocation of proteins and organelles that are damaged, denatured or aged to lysosomes for digestion and degradation [8]. This process not only inhibits tumour formation by limiting inflammation and eliminating toxic unfolded proteins but also removes damaged mitochondria that generate reactive oxygen species, causing DNA damage. Consequently, autophagy plays a vital role in the body's survival, differentiation and maintenance of homeostasis [9]. Specifically, autophagy plays a crucial role in maintaining cellular homeostasis under normal physiological circumstances [10]. It serves to prevent the buildup of harmful proteins and organelles during metabolic stress, thereby impeding cellular carcinogenesis [11, 12]. However, in the context of cancer, autophagy paradoxically contributes to tumour growth by supplying additional nutrients to cancer cells [13]. Consequently, autophagy exhibits a dual nature during the process of tumorigenesis and subsequent tumour development [14].

Numerous studies have investigated the involvement of autophagy in the development and advancement of PCa. Lu et al. [15] discovered that autophagy, triggered by the overexpression of DCTPP1, facilitates tumour progression and is indicative of unfavourable survival outcomes in PCa. The transcriptional regulation of key autophagy and lysosomal genes by androgen receptors can promote the progression of PCa [16]. Conversely, inhibiting SGK1‐induced autophagy may impede PCa metastasis by reversing epithelial‐mesenchymal transition (EMT), thereby enhancing the anti‐metastatic effects [17]. Therefore, it is of great value to study the role of the entire autophagy gene subset in the diagnosis and prognosis of prostate cancer.

Mendelian randomisation (MR) is an epidemiological technique that leverages genetic variation to evaluate the causal impact of a particular factor on an outcome, such as the risk of prostate cancer [18]. By virtue of genetic variants being randomly allocated during gamete formation and largely unaffected by environmental or lifestyle factors, MR offers reduced susceptibility to biases stemming from reverse causation and confounding variables. These distinctive attributes have positioned MR as a method akin to randomised controlled trials in terms of its comparability.

With the emergence of the big data era and advancements in high‐throughput sequencing, the analysis of autophagy‐related genes (ARGs) in PCa patients from publicly available databases has become feasible. Our study further encompasses the creation of a machine learning algorithm capable of accurately predicting the risk of PCa. Furthermore, by utilising GWAS data, we have substantiated a potential correlation between loci variation associated with prognostic genes and the progression of prostate hyperplasia to prostate cancer.

## 2 Methods

### 2.1 Acquisition of Transcriptome Data

The RNA expression profile of TCGA‐PRAD cohort (*n* = 496) from the TCGA database and the corresponding clinical data were selected to construct the prognostic model. In the GEO database, GSE54460 (*n* = 106) and MSKCC (*n* = 140, http://cbio.mskcc.org/cancergenomics/prostate/data/), were used as the validation group of RNA‐seq to check the stability and accuracy of the model. All data were converted to FPKM format, and log2 was converted for subsequent analysis. At the meantime, the chip data set in the GEO database was used, including GSE116918 (*n* = 248), GSE46602 (*n* = 36), GSE70768 (*n* = 111), GSE70769 (*n* = 92) prostate cancer chip data as a validation set. A shared characteristic of these validation cohorts is the inclusion of randomly assigned prostate cancer samples, each with a sufficiently large sample size. It is important to highlight that, due to the differing dynamic ranges and batch characteristics between bulk RNA‐seq and microarray data, we opted to analyse the aforementioned bulk datasets independently to ensure an equitable evaluation of our model, rather than performing an integrative analysis. The normalizeBetweenArrays function in the limma R package is used to correct the chip data. The data utilised for immunotherapy was IMvigor210 (in addition to the TIDE online predictions), and the data came from the IMvigor210CoreBiologies R package.

### 2.2 Processing the scRNA‐Seq Data

The scRNA‐seq data were obtained from the GSE193337 dataset in the GEO database. This dataset consisted of four normal/benign samples and four tumour samples. Data analysis was conducted using Seurat R package. During the process of cell quality control, it was ensured that the mitochondrial content remained below 20%. Cells with a detected gene number < 200 or > 9000 or a high mitochondrial transcript ratio (> 15%) were excluded. In this study, the data was normalised and highly variable genes (2000) were selected. Data transformation was performed to eliminate the effect of the cell cycle, using the parameter vars.to.regression = c(“S.Score”, “G2M.Score”). The functions NormalizeData, FindVariableFeatures and ScaleData from the Seurat package were utilised. After normalisation and scaling, the batch effect between patients was then removed using the harmony algorithm in Seurat. Subsequently, dimensionality reduction techniques UMAP and t‐SNE, as well as the Louvain clustering algorithm, all from Seurat, were employed. The FindAllMarkers function was used to calculate differential genes between clusters or cell types, with a significance threshold of adjusted *p* < 0.05, |log2FC| > 0.25 and expression ratio > 0.1.

### 2.3 Consensus Clustering Analysis

Autophagy‐related genes were obtained from the HADb database (Clustering | Autophagy database). Univariate Cox analysis was conducted on ARGs, resulting in the identification of genes with significant prognostic effects (*p* < 0.05). Utilising a resampling‐based technique known as consistent clustering, the TCGA‐PRAD cohort was subjected to cluster discovery using a set of genes with prognostic effects. This clustering process was executed using the ConsensusClusterPlus R package. Subsequently, the consensus score matrix, cumulative distribution function (CDF) curve and proportion of ambiguous clustering (PAC) score were employed to determine the most suitable number of clusters.

### 2.4 Cell Annotation Analysis

Cell type annotation was conducted using the SingleR package (v1.2.4) and subsequently verified through manual inspection. We visualised various cell markers including epithelial cell marker, fibroblast marker, endothelial cell marker, T cell marker, NK cell marker, B cell marker, myeloid cell marker and mast cell marker. Subsequently, we isolated and subjected epithelial cells, T cells and fibroblasts to cluster analysis and investigate their heterogeneity.

### 2.5 Subgroup Analysis of Each Cell Group

The Seurat's conventional methodology was employed to effectively isolate epithelial cells, T cells and fibroblasts for the purpose of discerning distinct subgroups. Specific markers were utilised to identify the subgroups within the larger group, and the utilisation of UMAP was demonstrated. The subclass analysis of T cells was annotated using ScType software.

CNV analysis of epithelial cells was conducted using the InferCNV software, with normal‐derived epithelial cells serving as a reference, to primarily detect malignant cells within tumour cell subsets. Pseudotime analysis of epithelial cells was performed using the monocle2 software. The dimension reduction algorithm employed DDRTree, while default parameters were utilised for the remaining steps, aiming to elucidate the cellular differentiation process.

### 2.6 Transcription Factor Analysis

To explore the dominant transcription factor in different subclones, we used the decouple R software for transcriptional factor analysis of each cell subgroup. This allows activity scores to be calculated on a cell‐by‐cell basis, describing how much each cell is enriched in TF and its downstream targets (regulators).

### 2.7 Immune Infiltration Analysis

The immune infiltration analysis of the TCGA‐PRAD dataset employed the ESTIMATE algorithm to compute the StromalScore, ImmuneScore, ESTIMATEScore and TumorPurity. Furthermore, CIBERSORT was utilised to assess the immune cell composition in prostate cancer. The Wilcoxon test was employed as the statistical method for comparing groups, with significance determined when *p* < 0.05.

To evaluate the efficacy of immunotherapy/chemotherapy, we employed the oncoPredict package in R to compute the semi‐inhibited concentration (IC50) of conventional chemotherapy drugs. Additionally, we utilised TIDE online (http://tide.dfci.harvard.edu/) to forecast the immune response and score of the TCGA dataset. The ggcorrplot package was employed to conduct a correlation analysis between risk scores and immune checkpoint expression.

### 2.8 GSEA Analysis and SNV Analysis

To assess the functional attributes of various tumour types, we employed the clusterProfiler R package to conduct Gene Set Enrichment Analysis (GSEA) on the differentially up‐regulated genes associated with each tumour type. Specifically, we enriched the Hallmark‐related gene set, which is a signature of the MSigDB database, when the *p* value of the Benjamini‐Hochberg correction was less than 0.05. To visualise the functional enrichment, we utilised the enrichplot R package.

Additionally, we obtained the SNV mutation data from the TCGA database and employed the maftools package to identify the Tumour Mutation Burden (TMB), Mutational Annotation and Thermodynamic (MATH) score and Homologous Recombination Deficiency (HRD). Subsequently, we performed differential analysis on the risk groups, employing the Wilcoxon test as the statistical method for assessing differences.

### 2.9 Establishment of Risk Model Generated by Machine Learning Algorithms

The DESeq2 R package was utilised to compute differentially expressed genes (DEGs) across various tumour types. Subsequently, single‐factor Cox analysis was employed to identify prognostic genes (*p* < 0.05) among the DEGs and autophagy‐related genes in each of the seven datasets, including TCGA‐PRAD. Genes exhibiting prognostic significance in at least six datasets were selected for further analysis. To enhance the precision and stability performance of the AIDPS model, we incorporated 10 machine learning algorithms and several algorithm combinations. These encompass random survival forest (RSF), elastic network (Enet), Lasso, Ridge, stepwise Cox, CoxBoost, Cox partial least squares regression (plsRcox), supervised principal component (SuperPC), generalised boosting regression model (GBM) and survival support vector machine (survival‐SVM). The signature generation procedure is as follows: (a) Univariate Cox regression analysis identified prognostic genes in seven datasets including TCGA‐PRAD (described in the previous step); (b) Then, 101 algorithm combinations were performed on prognostic genes to fit the prediction model based on the leave‐one‐out cross‐validation (LOOCV) framework in the TCGA‐PRAD cohort; (c) All models were detected in six validation datasets (GSE54460, MSKCC, GSE116918, GSE46602, GSE70768, GSE70769); (d) For each model, the Harrell Consistency Index (C‐index) is calculated in all validation data sets, and the model with the highest average C‐index is considered to be the best. Prognostic models were constructed using 101 machine learning methods, enabling the algorithm to assign a risk score to each patient. The surv_cutpoint was employed to determine the cutoff value for grouping. Patients were divided into high‐risk and low‐risk group.

### 2.10 SMR And MR Analysis

The GWAS data for prostate cancer were obtained from the GTCA website [GCTA | Yang Lab (westlake.edu.cn)]. The Manhattan plot of the GWAS was generated using the CMplot package. Gene colocalisation analysis was conducted using the SMR software, utilising a European reference population consisting of thousands of genomes and incorporating eQTLGen data (eQTLGen—cis‐eQTLs) and prostate cancer GWAS data. Mendelian randomisation analysis (MR) can estimate the causal effect of risk factors on complex diseases using genetic variation as instrumental variable (IV). The inverse‐variance weighted (IVW) method was used as the primary analysis. It was performed in this study using the R package TwoSampleMR, primarily to investigate the potential association between enlarged prostate and prostate cancer based on SNP sites corresponding to genes implicated in the prognostic model. The intercept value in MR‐Egger was used to evaluate pleiotropy. The intercept value in MR‐Egger was used to evaluate pleiotropy. If the intercept term was very close to 0, then the MR‐Egger regression model was very close to IVW.

### 2.11 Statistical Analysis

All data processing, statistical analysis and plotting were conducted using R 4.1.3 software. The correlation between two continuous variables was assessed using the Pearson correlation coefficient. Categorical variables were compared using the Chi‐square test, while continuous variables were compared using either the Wilcoxon rank sum test or *T*‐test. The optimal cutoff value was determined using the survminer package. Cox regression and Kaplan–Meier analysis were performed using survival kits. The CompareC package was utilised to compare c‐indexes for different variables.

## 3 Results

### 3.1 scRNA‐Seq Expression Profile of PRAD Cohorts

**FIGURE 1:**
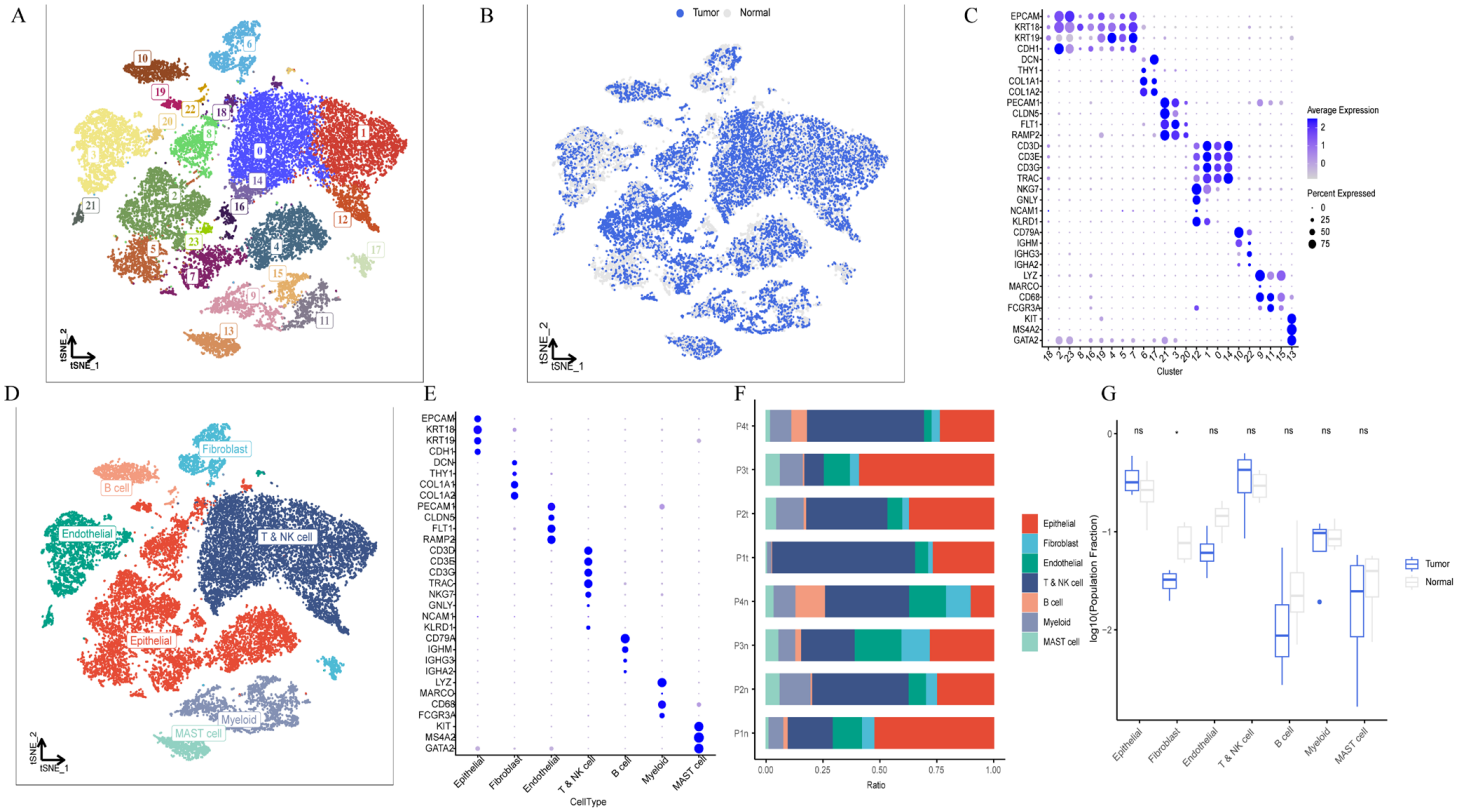
ScRNA‐seq analysis of PRAD. (A) tSNE plots of cluster classification in PRAD. (B) tSNE plots of the distribution of tumour and normal cells. (C) Bubble diagram of marker genes in each cluster. (D) tSNE plots of seven cell types. (E) Bubble diagram of marker genes in each cell type. (F) Bar chart of the ratio of each cell type. (G) Boxplot of cell composition of tumour and normal group.

After performing dimensionality reduction cluster analysis, a total of 24 clusters are obtained in Figure 1A. We observed the distribution of tumour cells and normal cells on the tSNE diagram (Figure 1B). Marker genes in each cluster are visualised in Figure 1C. According to the expression of marker genes, each cluster was annotated as seven cell types (Figure 1D). We identified epithelial cell markers (“EPCAM”, “KRT18”, “KRT19”, “CDH1”); Fibroblast marker (“DCN”, “THY1”, “COL1A1”, “COL1A2”); Endothelial cell marker (“PECAM1”, “CLDN5”, “FLT1”, “RAMP2”); T cell marker (“CD3D”, “CD3E”, “CD3G”, “TRAC”); NK cell maker (“NKG7”, “GNLY”, “NCAM1”, “KLRD1”); B cell marker (“CD79A”, “IGHM”, “IGHG3”, “IGHA2”); Myeloid cell marker (“LYZ”, “MARCO”, “CD68”, “FCGR3A”) and mast cell marker (KIT, MS4A2, GATA2) in Figure 1E. There was a significant difference in the content of fibroblasts between normal and tumour samples (Figure 1F,G).

### 3.2 Subclassification Analysis, Pseudotime Analysis and CNV Analysis of Epithelial Cells

**FIGURE 2:**
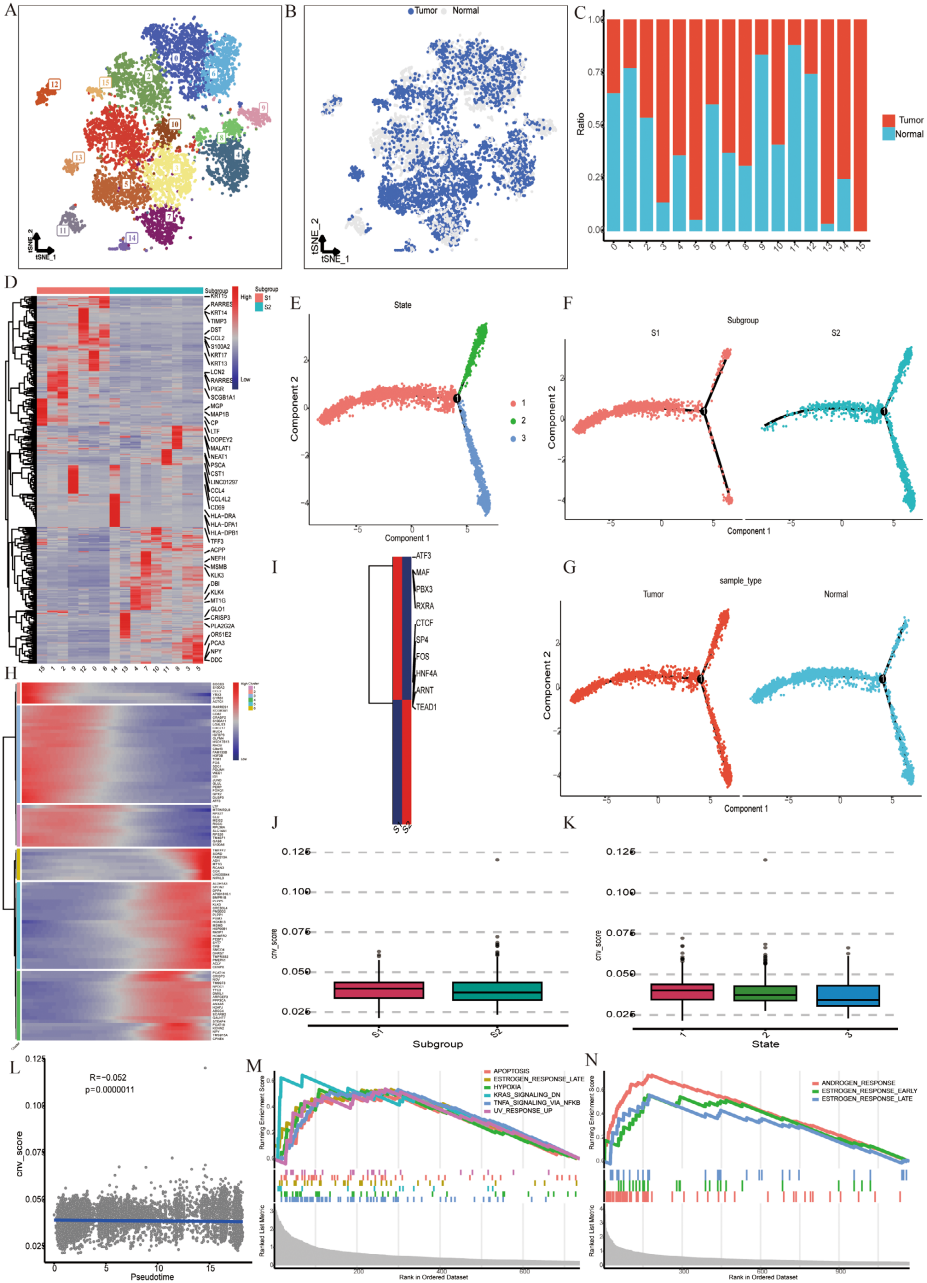
Subclassification analysis, pseudotime analysis and CNV analysis of epithelial cells. (A) tSNE plot of epithelial cells by dimension reduction cluster analysis. (B) tSNE plot of the distribution of tumour and normal epithelial cells. (C) Bar chart of the ratio of tumour and normal epithelial cells in each cluster. (D) Heatmap of top 30 DEGs between S1 and S2. (E–G) Pseudotime analysis of epithelial cells. (H) Heatmap of DEGs between clusters. (I) Transcription factor analysis of epithelial cells. (J–L) CNV analysis of epithelial cells. (M, N) GSEA pathway analysis of epithelial cells.

The epithelial cells were isolated for dimensional reduction cluster analysis. The tSNE diagram showed 16 clusters and the distribution of tumour and normal cells (Figure 2A,B). It was found that tumour cells accounted for the vast majority in c3,4,5,7,8,13,14 and 15, while normal epithelial cells accounted for a relatively low proportion in these clusters (Figure 2C). Top 30 DEGs of each cluster are identified in Figure 2D. c15,1,2,9,12,0 and 6 were clustered together with similar functions and named as subgroup1 (S1). The remaining clusters were grouped together and named as subgroup2 (S2). We performed cell trajectory analysis on epithelial cells in Figure 2E–G. It could be seen that State1 (normal cells) might be the starting point for tumour differentiation, branching into two types State2 or State3, and S1 was mostly normal cells, which were enriched in State1 and State2. Most of S2 are tumour cells, which were enriched in State2 and State3. We also conducted CNV analysis of cells in each State and found that there was not significant difference in CNV score among three states. Pearson correlation analysis was conducted on CNV score and pseudotime, and the two groups were positively correlated (*R* = −0.052, *p* = 0.0000011) (Figure 2J–L), indicating that there was little difference in CNV between benign and malignant tissues. Transcription factor analysis results indicated that S1 was enriched in ATF3, MAF, PBX3, RXRA and CTCF, and S2 was enriched in SP4, FOS, HNF4A, ARNT and TEAD1 (Figure 2I). In the GSEA pathway analysis, S1 was related to APOPTOSIS, ESTROGEN_RESPONSE_LATE, HYPOXIA, KRAS_SIGNALING_DN, TNFA_SIGNALING_VIA_NFKB and UV_RESPONSE_UP. S2 was associated with ANDROGEN_RESPONSE, ESTROGEN_RESPONSE_EARLY and ESTROGEN_RESPONSE_LATE (Figure 2M,N).

### 3.3 Subclassification Analysis of T Cells and Fibroblasts

**FIGURE 3:**
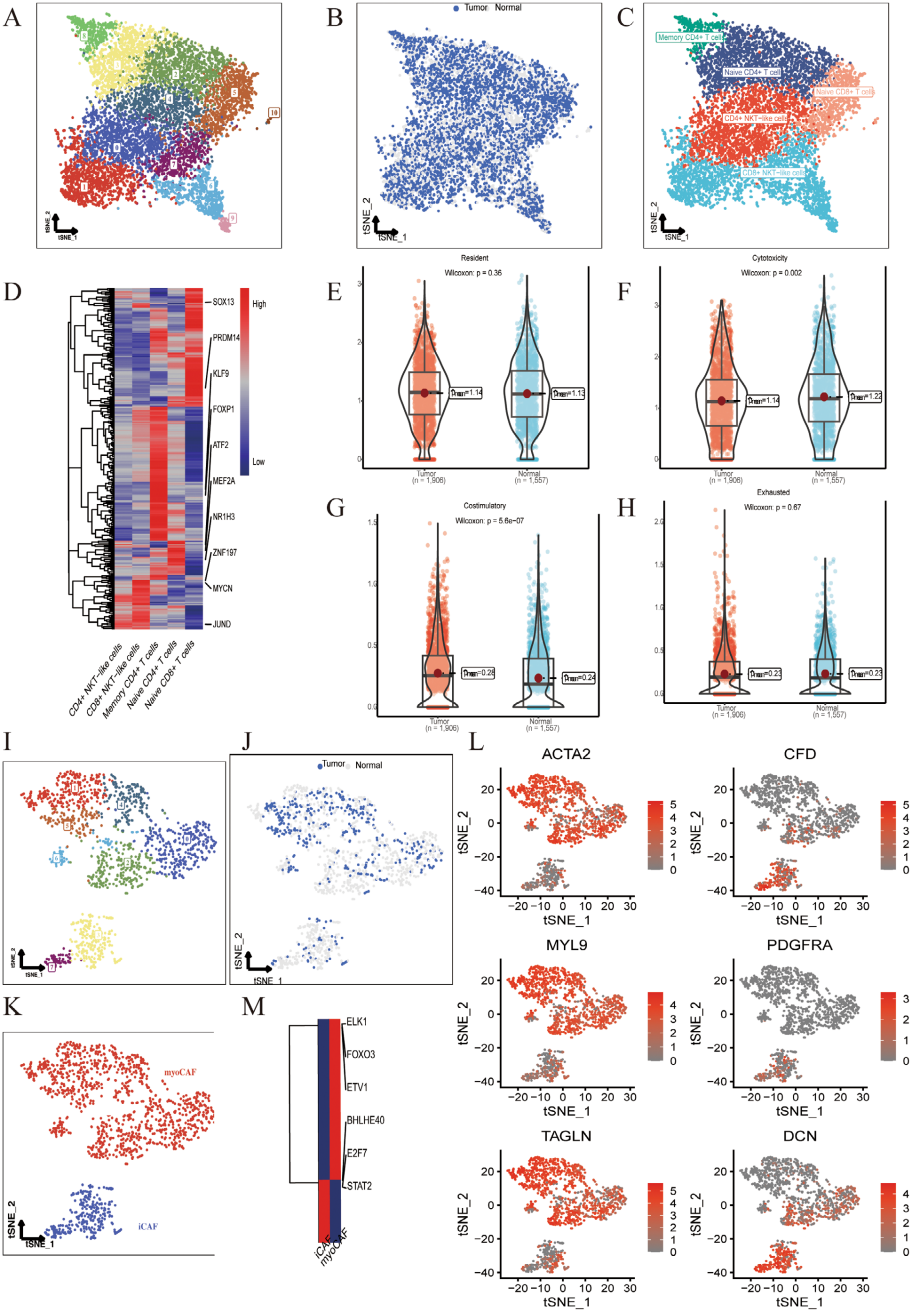
Subclassification analysis of T cells and fibroblasts. (A) tSNE plot of T cells by dimension reduction cluster analysis. (B) tSNE plot of the distribution of tumour and normal T cells. (C) tSNE plots of cell subtypes. (D) Transcription factor analysis on T cell types. (E–H) Resident, cytotoxic, exhausted and costimulatory scores of CD8+ T cells between tumour and normal group. (I) tSNE plot of fibroblasts by dimension reduction cluster analysis. (J) tSNE plot of the distribution of tumour and normal fibroblasts. (K) tSNE plot of the distribution of myoCAF and iCAF. (L) tSNE plots of marker genes in fibroblasts. (M) Transcription factors of myoCAF and iCAF.

T cells were isolated for dimensional reduction cluster analysis and 11 clusters were shown in Figure 3A. The distribution of tumour and normal cells was visualised in the tSNE diagram (Figure 3B). Each cell type is annotated in Figure 3C, including memory CD4+ T cells, naïve CD4+ T cells, naïve CD8+ T cells, CD4+ NKT‐like cells and CD8+ NKT‐like cells. We performed transcription factor analysis on various T cell types (Figure 3D). Naive CD8+ T cells were enriched in SOX13 and PRDM14, memory CD4+ T cells were enriched in KLF9 and FOXP1, and naive CD4+ T cells were enriched in NR1H3 and MEF2A. CD8+ NKT‐like cells were enriched in ZNF197 and MYCN, and CD4+ NKT‐like cells were enriched in ATF2 and JUND. Resident, cytotoxic, exhausted and costimulatory scores of CD8+ T cells were calculated, and it was found that scores of tumour group were significantly higher than those of normal group (Figure 3E–H).

Subsequently, we isolated the fibroblasts. The tSNE diagram showed eight clusters from fibroblasts (Figure 3I). We understood the distribution of tumour and normal cells from Figure 3J. We classified fibroblasts into myofibroblasts (myoCAF) and inflammatory phenotypes (iCAF) in Figure 3K. Subgroup 3 and 7 expressed inflammatory subtypes such as CFD, PDGFRA and DCN, and the remaining tumour cells expressing inflammatory markers were named iCAF. The remaining subgroups were identified as myofibroblasts CAFs (myoCAF) because of upregulation of myofibroblast markers, including alpha smooth muscle actin (alpha SMA, also known as ACTA2) and contractile proteins (TAGLN, MYLK, MYL9) (Figure 3L). Two CAFs were enriched in different transcription factors. iCAF enriched in BHLHE40, E2F7, STAT2 and myoCAF enriched in ELK1, FOXO3 and ETV1 (Figure 3M).

### 3.4 Collection of Autophagy‐Related Prognostic Gene Sets

**FIGURE 4:**
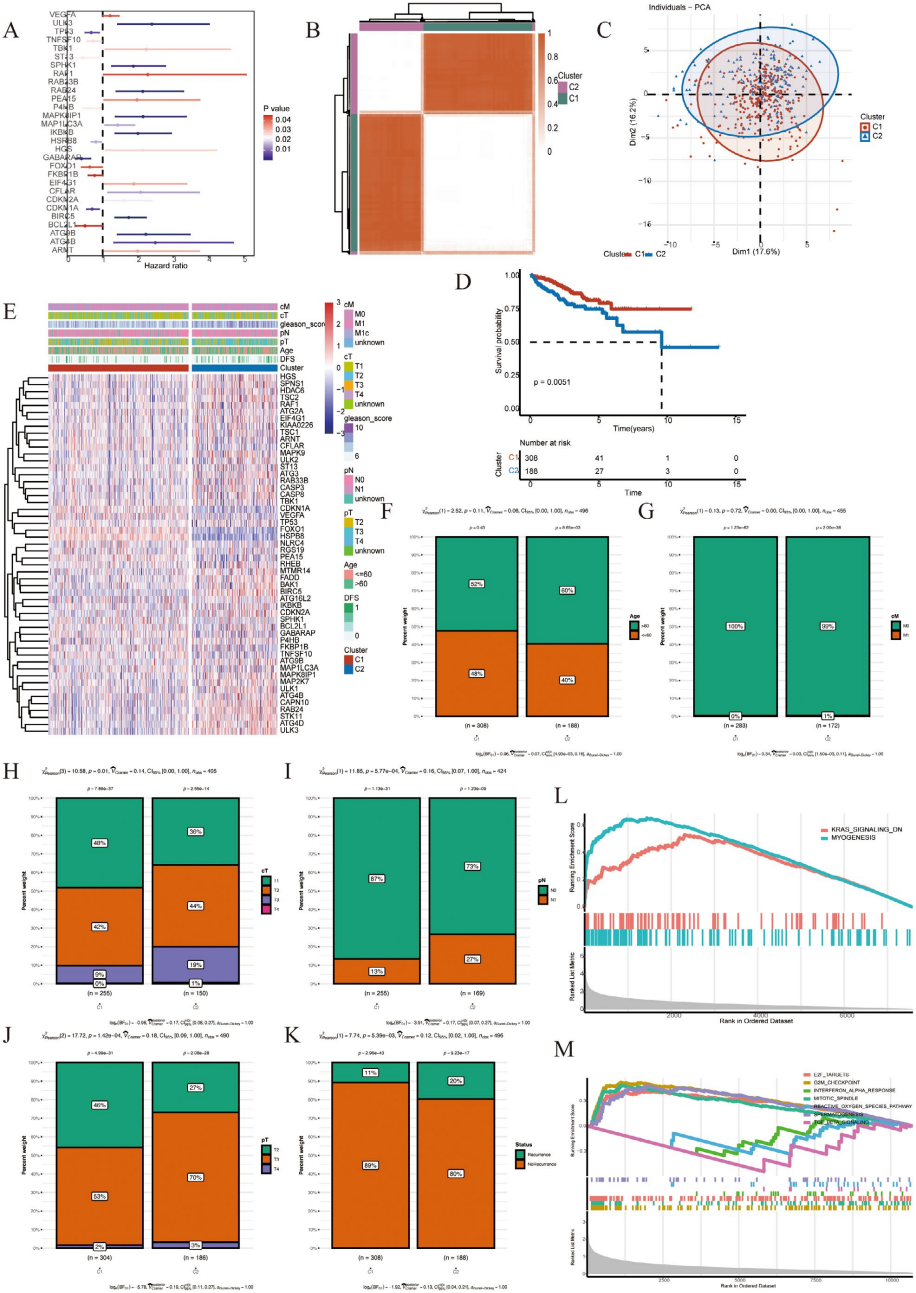
Collection of autophagy‐related prognostic gene sets. (A) Identification of prognostic genes. (B) Heat map of consistent cluster analysis in PRAD patients. (C) PCA analysis of C1 and C2. (D) K–M curve of C1 and C2. (E) Heat map of expression level of prognostic genes. (F–K) Bar chart of per cent weight of clinical indicators in two clusters. (L, M) GSEA analysis of C1 and C2.

Univariate Cox analysis was performed on 222 autophagy‐related genes (ARGs), and 29 genes with prognostic effect were screened out (*p* < 0.05, Figure 4A). Based on the expression of 29 prognostic genes, we performed a consistent cluster analysis in which all PRAD samples were initially grouped into clusters (*k* = 2–9). The ratio of the cumulative distribution function (CDF) curve of the consistency score matrix and principal component analysis (PAC) results indicated that the best number was obtained when *k* = 2, resulting in two cluster clusters (C1 and C2) (Figure 4B). Principal component analysis showed the distribution of samples for both types (Figure 4C). K–M curve analysis showed that C1 had a poorer prognosis than C2 (*p* = 6e‐4) in Figure 4D. According to the expression level of autophagy‐related genes, most genes were highly expressed in C1 (Figure 4E). Through Chi‐square test, it was found that there were significant differences between clinical indicators (cT, cM, pN, pT, age and recurrence) between C1 and C2 (*p* < 0.05) (Figure 4F–K). We conducted GSEA analysis for marker genes in C1 and C2 (Figure 4L,M).

### 3.5 Construction of Risk Model Generated by Artificial Intelligence‐Derived Prognostic Signature (AIDPS)

**FIGURE 5:**
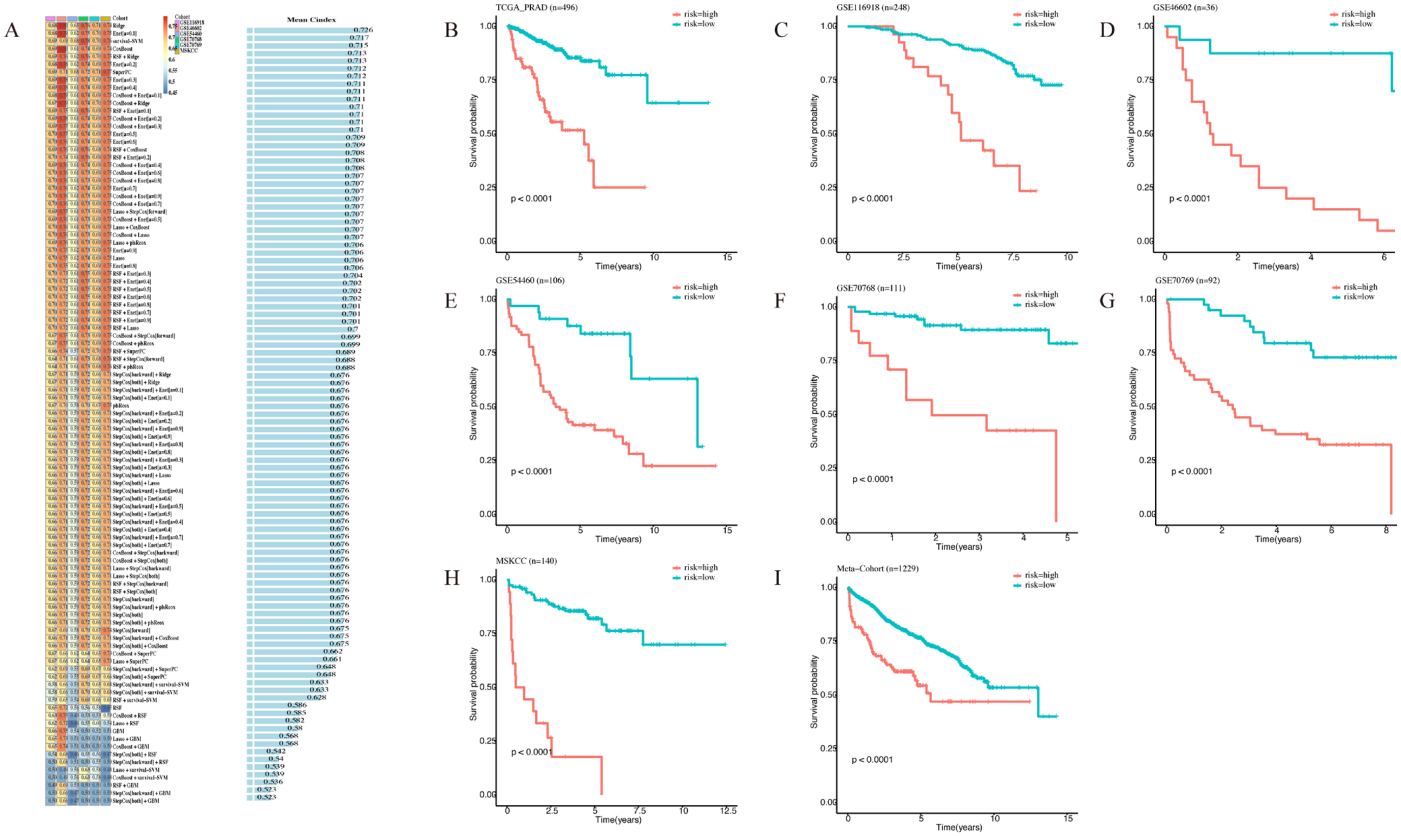
Construction of risk model generated by artificial intelligence‐derived prognostic signature (AIDPS). (A) Heat maps of average C‐index of six validated datasets in 101 machine learning prognostic models. (B–I) K–M curve analysis of TCGA training data and six validation datasets. (I) K–M curve analysis of meta‐cohort.

Differentially expressed genes and ARGs in each dataset (seven datasets including TCGA‐PRAD), univariate Cox analysis was used to screen out prognostic genes (*p* < 0.05). Genes that have a prognostic effect in at least six datasets will be identified for subsequent analysis. These genes went through our machine learning‐based integration program to develop artificial intelligence‐derived prognostic signature (AIDPS). In the TCGA‐PRAD dataset, we fit 101 prediction models through the LOOCV framework and further calculate the C‐index for each model in all validation datasets (Figure 5A). Interestingly, the best model is the Ridge, with the highest average C‐index (0.726), and this combined model has a leading C‐index across all validated datasets (Figure 5A). Next, all patients were divided into high‐risk group and low‐risk group according to the optimal threshold. As shown in the Figure 5B–I, in the TCGA‐PRAD dataset and the other six validated datasets, the disease‐free period (DFS) of patients in the high‐risk group was significantly worse than that in the low‐risk group (all *p* < 0.05). A meta‐cohort combined with all samples also showed the same trend (*p* < 0.05) (Figure 5J).

### 3.6 Evaluation of AIDPS

**FIGURE 6:**
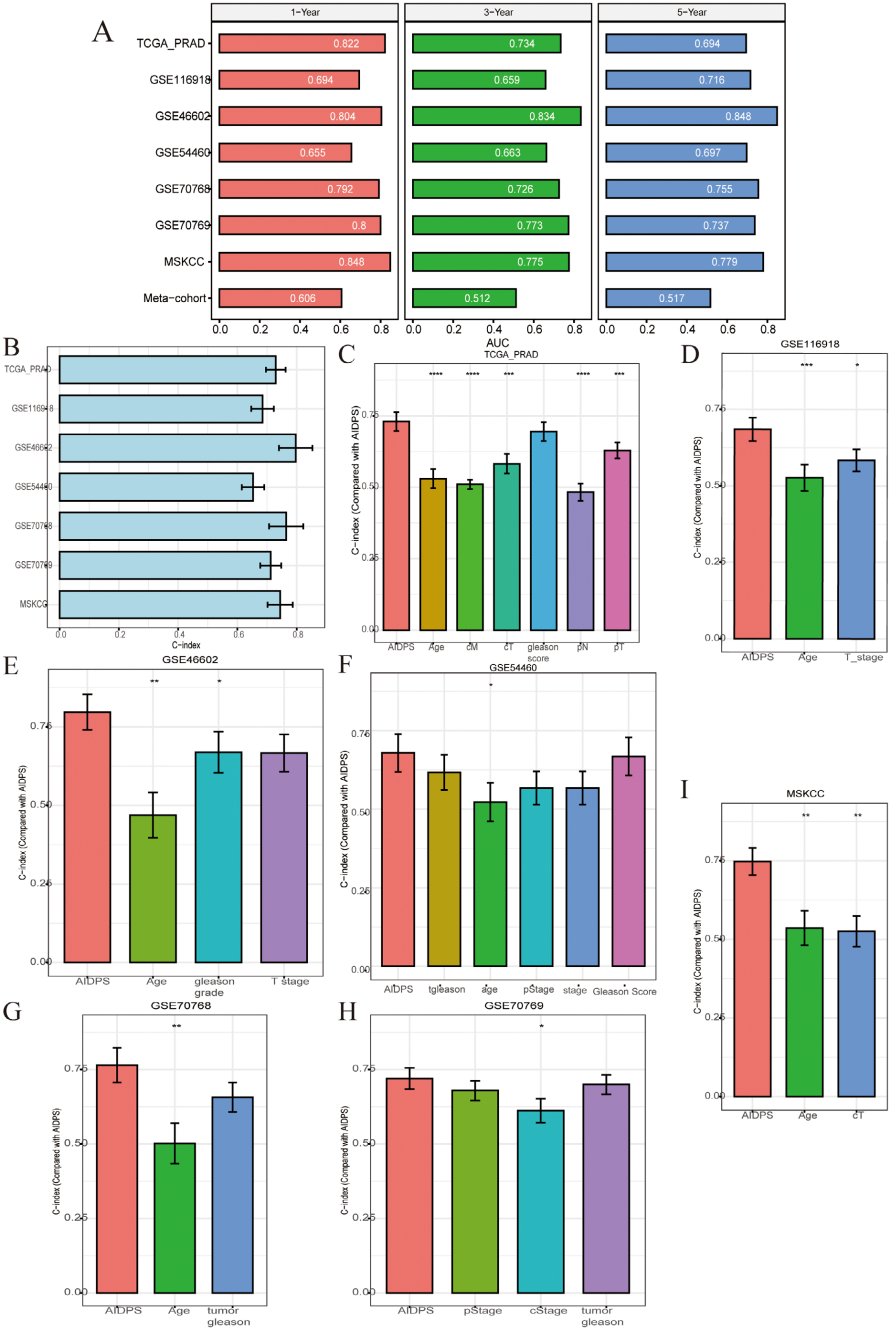
Evaluation of AIDPS. (A) Bar chart of AUC values for each dataset at 1, 3 and 5 years. (B) C‐index for each dataset. (C–I) C‐index for AIDPS and other clinical measures of TCGA training set and seven validation datasets. * *p* < 0.05, ** *p* < 0.01, *** *p* < 0.001, **** *p* < 0.0001.

ROC analysis measured the discrimination of AIDPS, and the 1‐, 3‐ and 5‐year AUC of TCGA‐PRAD were 0.822, 0.734 and 0.694, respectively (Figure 6A). We presented a bar chart of the C‐index (95% confidence interval) for each cohort (Figure 6B). All of these results indicated that AIDPS had a stable and robust performance in multiple independent cohorts. A previous study reported that clinical features, such as AJCC staging, were also used to assess the prognosis of PRAD in clinical practice. Therefore, we compared the performance of AIDPS with that of other clinics in predicting prognosis. As shown in Figure 6C–I, the accuracy of AIDPS was significantly better than that of other variables including age, sex, pathological T, N, M, gleason_score and Stage.

### 3.7 Comparison of the Prognostic Models From Other Studies

**FIGURE 7:**
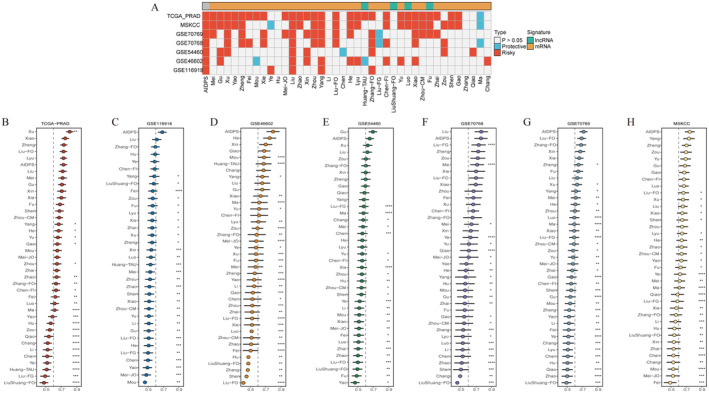
Comparison of the prognostic models from other studies. (A) Heat map of correlation analysis of AIDPS model in seven datasets. (B–H) C‐index of each model in seven datasets. * *p* < 0.05, ** *p* < 0.01, *** *p* < 0.001, **** *p* < 0.0001.

To compare the performance of AIDPS with other prognostic models, we conducted a comprehensive search of prognostic models around the published studies. Due to the severe lack of miRNA information in validation datasets of some chips, a prognostic model of miRNA was excluded. Finally, 41 prognostic models (including mRNA and lncRNA prognostic models) were collected. These characteristics were associated with various biological processes, such as immune response, autophagy, iron death, dryness, epithelio‐mesenchymal transformation, copper death, hypoxia, glycolysis, lipogenesis, vitamin D, epigenetics, N6‐methyladenosine, aging, WNT and drug sensitivity. We performed univariate Cox regression analyses for each prognostic model and all datasets and observed that our model was significantly associated with prognostic outcomes in almost all cohorts (Figure 7A), demonstrating the stability of AIDPS. In addition, the C‐index of AIDPS was compared with other characteristics. Notably, AIDPS showed better performance in every dataset than almost all models (Figure 7B–H). We noted that most models performed well in their own training dataset and some external datasets (e.g. Mei − JO, Zhai), but poorly in others. This may be due to poor generalisation of the resulting model from overfitting. Our model was dimensionally reduced by two machine learning algorithms with better extrapolation potential.

### 3.8 Predictive Response to Immunotherapy/Chemotherapy in the Risk Group

**FIGURE 8:**
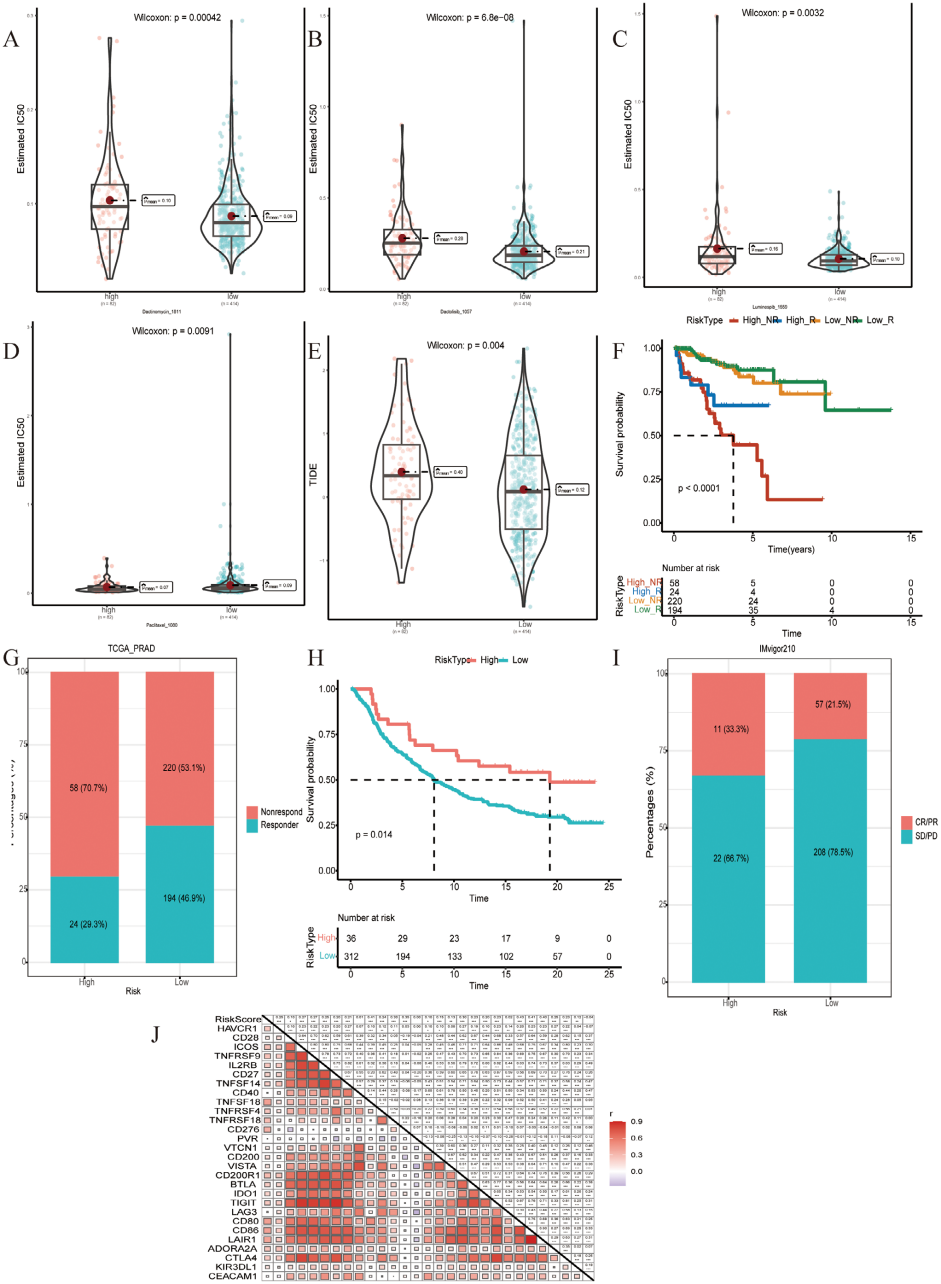
Predictive response to immunotherapy/chemotherapy in the risk group. (A–D) Violin charts of IC50 values of the chemotherapeutic drugs Dactinomycin_1811, Dactolisib_1057, Luminespib_1559 and Paclitaxel_1080 in high and low‐risk groups. (E) Violin plot of TIDE values in risk groups. (F) K–M curve analysis results of the risk‐immune response group. (G) Bar chart of proportion of immune responses in the risk group. (H) K–M curve analysis of the IMvigor210 dataset based on the AIDPS prognostic model. (I) Bar chart of the proportion of immune responses in the risk group in the IMvigor210 dataset. (J) Heat map of correlation between risk value and expression level of immune checkpoint.

We found significant differences in IC50 values of the Dactinomycin_1811, Dactolisib_1057, Luminespib_1559 and Paclitaxel_1080 between groups (Figure 8A–D). In TIDE online analysis, there was a significant difference in TIDE values between risk groups (Figure 8E). K–M curve analysis showed that the high‐risk no response group had a poorer prognosis (Figure 8F). The bar chart exhibited the proportion of responders and non‐responders in the risk group (Figure 8G). Using immunotherapy data IMvigor210, K–M curve analysis indicated that the low‐risk group had a worse prognosis than the high‐risk group (Figure 8H). The proportion of responders and non‐responders in the risk group was calculated based on the IMvigor210 dataset (Figure 8I). In addition, significant correlation between risk values and several immune checkpoints is visualised in Figure 8J.

### 3.9 SNV Mutations and Immune Infiltration

**FIGURE 9:**
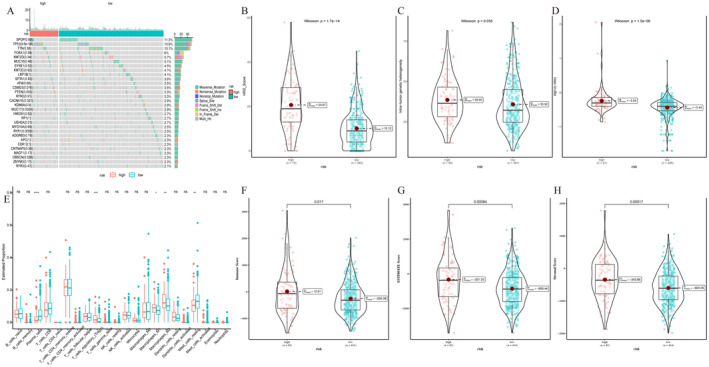
SNV mutations and immune infiltration. (A) The waterfall diagram of SNV mutation between two groups. (B–D) The difference of TMB, MATH and HRD scores between the two groups. (E) Box plot of the difference of 22 immune cells between the two groups based on CIBERSORT algorithm. (F–H) The difference of StromalScore, ImmuneScore and ESTIMATEScore between the two groups.

The waterfall map showed top 30 mutated genes in both risk groups. SPOP (11.3%), TP53 (10.9%) and TTN (10.1%) were the genes with high mutation rates, and TP53 was significantly different between the two groups (Figure 9A). We calculated the TMB, MATH and HRD scores to assess differences of SNV mutations between the two subtypes, and we found significant differences in TMB score between groups (*p* < 0.05) (Figure 9B–D). The StromalScore and ESTIMATEScore indexes had significant differences between risk groups (*p* < 0.05). StromalScore, ImmuneScore and ESTIMATEScore were higher in the high‐risk group (Figure 9F–H). In the immune infiltration analysis, it was found that Plasma_cells and Mast_cells_resting had a higher content in the low‐risk group. T_cells_regulatory_(Tregs) and Macrophages_M1, and Macrophages_M2 were higher in the high‐risk group (Figure 9E).

### 3.10 SMR And MR Analyses

**FIGURE 10:**
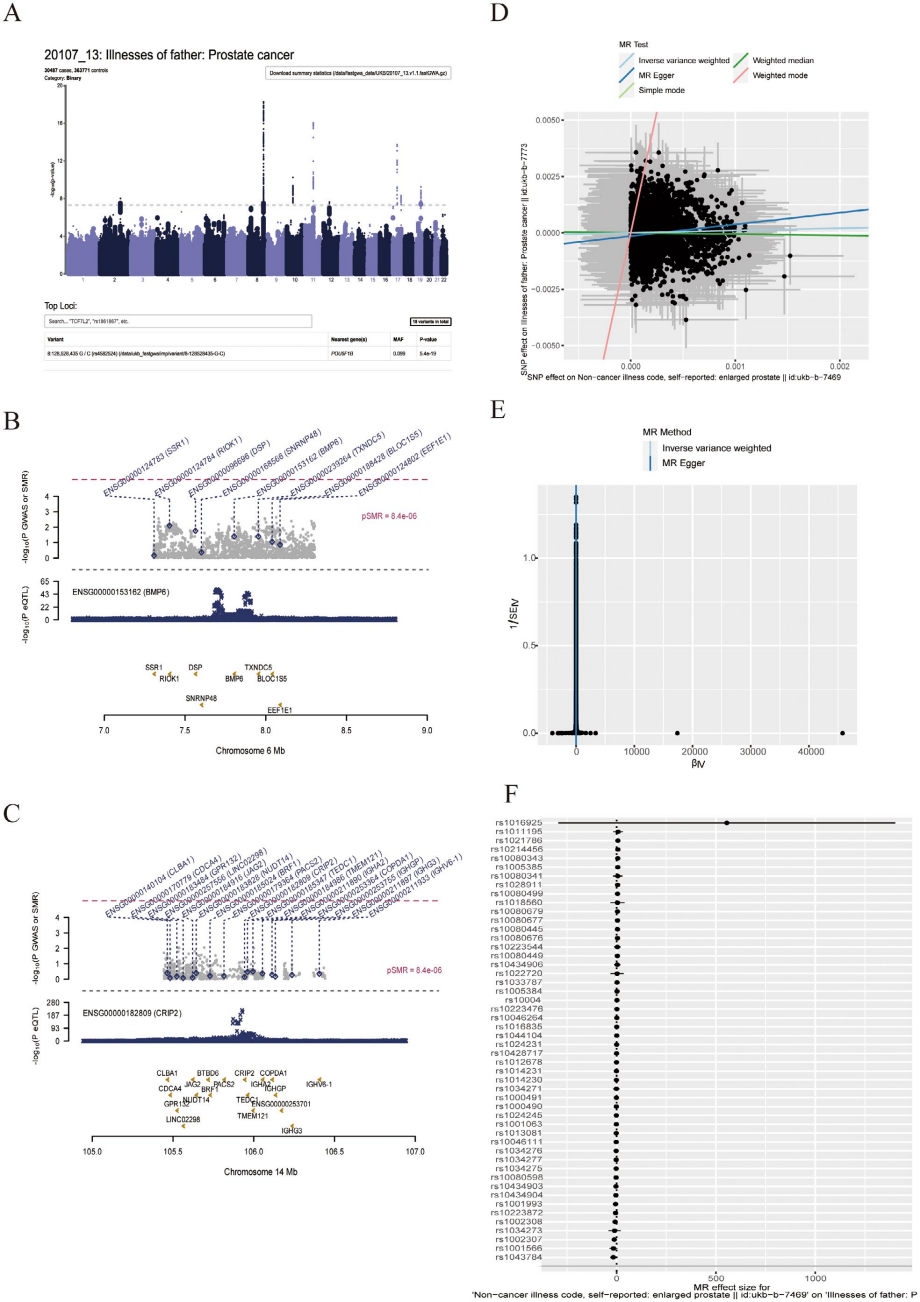
SMR and MR analyses. (A) Manhattan map of GWAS data for prostate cancer. (B, C) Results of gene colocalisation analysis of BMP6 and CRIP2. (D–F) The results of MR analysis of the SNP site of the BMP6 gene between prostatic hyperplasia (ukb‐b‐7469) and prostate cancer (ukb‐b‐7773).

GWAS data for prostate cancer were visualised using the Manhattan chart (Figure 10A). Using SMR software, thousands of Europeans with genomes as reference and eQTLGen data (EQTLgen‐CIS‐EQTLS) and prostate cancer GWAS data for gene colocalisation analysis, we showed the results of prognostic model‐related genes (BMP6 and CRIP2) (Figure 10B,C). Using Mendelian randomisation (MR) analysis, it is found that there was a significant correlation between prostate hyperplasia (ukb‐b‐7469) and prostate cancer (ukb‐b‐7773) in the SNP site of BMP6 gene based on the MR Egger algorithm. In SNP sites such as rs2743987, rs7768988, there was a significant correlation between prostate hyperplasia (ukb‐b‐7469) and prostate cancer (ukb‐b‐7773) in enlarged_prostate_to_Prostate_cancer_mr_snps_result.xlsx, Figure 10D–F.

## 4 Discussion

Prostate cancer is a clinically heterogeneous disease with the genetic heterogeneity [19]. The stroma in the normal prostate mainly consists of smooth muscle cells while it is partially replaced by fibroblasts and myofibroblasts during carcinomatous change [20]. In current study, we found that there were significant differences in the content of fibroblasts between normal samples and tumour samples from the perspective of cell type composition as previous results. In other words, during tumour evolution, distinct tumour subclones acquire preferential transcriptional subtypes which show different cellular differentiated status. An ongoing accumulation of mutations results in increased genetic diversity, with the tumour acquiring distinct subclones. In addition to cell proliferation status and expression program, copy number aberration can also be inferred from scRNA‐seq data. scRNA‐seq analysis provides a feasible strategy for mapping developmental states to tumour subcloning evolution, which may help to understand collective behaviour and regulatory mechanisms within the tumour ecosystem [21]. Therefore, it was necessary to evaluate tumour cell transcriptional status among different tumour subclones in untreated PCa patients at the single‐cell level, in order to support that transcriptional heterogeneity may also play an important role in cancer progression, adaptation and evolution within the tumour ecosystem [22, 23].

The epithelial cells, T cells and fibroblasts were isolated and clustered to explore the heterogeneity of PCa. In epithelial cells, S1 was enriched in transcription factors ATF3, MAF, PBX3, RXRA and CTCF, and S2 was enriched in SP4, FOS, HNF4A, ARNT and TEAD1. iCAF was enriched in BHLHE40, E2F7, STAT2, and myoCAF was enriched in ELK1, FOXO3 and ETV1. These transcription factors play a crucial role in recognising specific DNA sequences to regulate chromatin structure and transcription processes, thereby influencing the proliferation and invasion of cancer cells. For instance, ATF3 has been reported to induce apoptosis and inhibit the outgrowth of prostate cancer, while simultaneously promoting the invasion of prostate cancer cells [24]. In this context, gene regulatory networks can elucidate the mechanisms underlying cancer progression and metastasis, positioning transcription factors as promising targets for therapeutic intervention.

Autophagy exhibits a dual function in oncogenesis, acting both as a promoter and suppressor of cancer. It has been reported in a variety of cancers concerning the role of autophagy in tumorigenesis, progression and therapeutic resistance [25], such as glioma, breast cancer, hepatocellular carcinoma. Previous studies have shown that cell migration‐inducing protein (CEMIP) accelerates the protective autophagy in PCa cells [26]. However, most studies are lacking to signal ARGs for the prognosis of PCa. In this analysis, we identified important prognostic ARGs and several DEGs for further establishment of predictive models based on machine learning methods. In recent years, machine learning has been applied to predict and prognose cancer [27]. Current prognostic models for PCa frequently depend on subjective choices of modelling algorithms and often lack validation across multiple datasets, resulting in suboptimal performance or model overfitting [47]. To mitigate these issues, we compiled an extensive array of commonly employed machine learning algorithms and developed 101 distinct models. Many different machine learning tasks are associated with each characteristic of cancers [28]. The best‐performing machine learning method is the Ridge, with the highest average C‐index (0.726). To tailor the regularisation strength to individual feature spaces, ridge regression has been advanced to banded ridge regression, which optimises distinct regularisation hyperparameters (alpha) for each feature space. It is the first study to develop AIDPS for PCa in the prognosis and treatment of this disease. To standardise the process of benchmarking, we unified existing benchmarking datasets and defined a rigorous set of evaluation metrics. Additionally, we found that the prognostic model AIDPS was the most effective compared to other 41 published models. AIDPS showed better performance in each dataset than almost all models with the higher C‐index value. The C‐index reflected that AIDPS was the most powerful signature than other models and traditional clinical parameters in other multicenter PCa cohorts, revealing the robustness in prognostic prediction. In the field of drug sensitivity and therapeutic response, the IC50 values of Dactinomycin_1811, Dactolisib_1057, Luminespib_1559 and Paclitaxel_1080 were significantly different in the high and low‐risk groups. In the TIDE online analysis, there were significant differences in TIDE values between the two risk groups. It indicates that the AIDPS model can be used to guide clinical medication. Recent advancements in sequencing technology, particularly single‐cell RNA sequencing (scRNA‐seq), in conjunction with machine learning methodologies, have yielded significant insights into the intricate landscape of various multi‐gene panels and their roles in cancer progression. Consequently, our AIDPS signature provides valuable utility as an adjunct tool for assessing prostate cancer prognosis and stratifying patients into high‐ or low‐risk categories for aggressive disease.

We also observed a higher mutation frequency in the risk groups with significantly prevalent TP53 mutation between groups. Previous studies have indicated that mutations in TP53 contribute to the proliferation and metastasis of PCa [29]. It substantiates the poorer prognosis observed in the high‐risk group. Finally, we selected two prognostic model‐related genes (BMP6 and CRIP2) with significance. Bone metastasis of prostate cancer acquired resistance to androgen deprivation through WNT5A‐mediated BMP‐6 induction [30]. Through performing MR analysis, it is found that there was a significant correlation between prostate hyperplasia and prostate cancer in the SNP site of BMP6 gene based on the MR Egger algorithm, such as rs2743987, rs7768988. BMP‐6 can drive the progression of prostate cancer [31]. The presence of Crip2 has been observed in the endothelial cells of the neovasculature during the processes of wound healing and tumour growth [32]. Previous study demonstrated the role of Atox1 in facilitating the transfer of copper to CRIP2, leading to a consequential alteration in CRIP2's secondary structure, ultimately resulting in the promotion of its degradation through the ubiquitin‐mediated proteasomal pathway [33]. MDA‐MB‐231 cells that underwent stable transfection with CRIP2 exhibited notable decreases in cell viability, migration and invasion, alongside diminished tumour growth and angiogenesis in mouse xenograft models [34]. The potential function of these two genes should be further explored in the mechanisms of PCa.

However, some limitations cannot be ignored. Firstly, the expression profile and clinical data of this study were downloaded from the public database. Prospective and multicenter studies are needed to validate our findings in the future. Secondly, there is a lack of basic experiments to explore the molecular mechanism of ARGs regulating the occurrence and progression of PCa. The clinical applicability of AIDPS remains limited due to inadequate incorporation of machine learning algorithms and underutilisation of existing data. Further clinical practice is also necessary for AIDPS in the prognosis of PCa. Overall, we developed an autophagy‐related model and tested its effectiveness using multiple GEO datasets, which is helpful for clinical decision‐making.

## Author Contributions

**Zhiyi Zhao:** conceptualization (lead), data curation (lead), formal analysis (lead), software (lead), visualization (lead), writing – original draft (lead), writing – review and editing (lead). **Yongjin Yang:** supervision (equal). **Zhou Sun:** data curation (equal), visualization (equal). **LianMing Fan:** data curation (equal). **Lingyun Liu:** conceptualization (equal), supervision (lead).

## Ethics Statement

The authors have nothing to report.

## Consent

The authors agree for publication.

## Conflicts of Interest

The authors declare no conflicts of interest.

## References

[1] S. A. Rosenthal , C. Hu , O. Sartor , et al., “Effect of Chemotherapy With Docetaxel With Androgen Suppression and Radiotherapy for Localized High‐Risk Prostate Cancer: The Randomized Phase III NRG Oncology RTOG 0521 Trial,” Journal of Clinical Oncology 37, no. 14 (2019): 1159–1168. 30860948 10.1200/JCO.18.02158 PMC6506419

[2] N. Tan , W. C. Lin , P. Khoshnoodi , et al., “In‐Bore 3‐T MR‐Guided Transrectal Targeted Prostate Biopsy: Prostate Imaging Reporting and Data System Version 2‐Based Diagnostic Performance for Detection of Prostate Cancer,” Radiology 283, no. 1 (2017): 130–139. 27861110 10.1148/radiol.2016152827 PMC5375629

[3] J. M. Genkinger , K. Wu , M. Wang , et al., “Measures of Body Fatness and Height in Early and Mid‐To‐Late Adulthood and Prostate Cancer: Risk and Mortality in the Pooling Project of Prospective Studies of Diet and Cancer,” Annals of Oncology 31, no. 1 (2020): 103–114. 31912782 10.1016/j.annonc.2019.09.007 PMC8195110

[4] S. A. Kenfield , M. J. Stampfer , J. M. Chan , and E. Giovannucci , “Smoking and Prostate Cancer Survival and Recurrence,” JAMA 305, no. 24 (2011): 2548–2555. 21693743 10.1001/jama.2011.879 PMC3562349

[5] J. Guan , X. Jiang , Y. Guo , et al., “Autophagy Inhibition and Reactive Oxygen Species Elimination by Acetyl‐CoA Acetyltransferase 1 Through Fused in Sarcoma Protein to Promote Prostate Cancer,” BMC Cancer 22, no. 1 (2022): 1313. 36517760 10.1186/s12885-022-10426-5 PMC9753422

[6] R. G. Cremers , K. K. Aben , I. M. van Oort , et al., “The Clinical Phenotype of Hereditary Versus Sporadic Prostate Cancer: HPC Definition Revisited,” Prostate 76, no. 10 (2016): 897–904. 26989049 10.1002/pros.23179 PMC5069637

[7] M. McIntosh , M. J. Opozda , M. O'Callaghan , A. D. Vincent , D. A. Galvão , and C. E. Short , “Impact of Different Unconditional Monetary Incentives on Survey Response Rates in Men With Prostate Cancer: A 2‐Arm Randomised Trial,” BMC Medical Research Methodology 22, no. 1 (2022): 252. 36175831 10.1186/s12874-022-01729-z PMC9520096

[8] S. Paik , J. K. Kim , P. Silwal , C. Sasakawa , and E. K. Jo , “An Update on the Regulatory Mechanisms of NLRP3 Inflammasome Activation,” Cellular and Molecular Immunology 18, no. 5 (2021): 1141–1160. 33850310 10.1038/s41423-021-00670-3 PMC8093260

[9] B. Hu , Y. Zhang , L. Jia , et al., “Binding of the Pathogen Receptor HSP90AA1 to Avibirnavirus VP2 Induces Autophagy by Inactivating the AKT‐MTOR Pathway,” Autophagy 11, no. 3 (2015): 503–515. 25714412 10.1080/15548627.2015.1017184 PMC4502722

[10] Y. Sun , H. Zou , L. Yang , et al., “Effect on the Liver Cancer Cell Invasion Ability by Studying the Associations Between Autophagy and TRAP1 Expression,” Oncology Letters 16, no. 1 (2018): 991–997. 29963174 10.3892/ol.2018.8774 PMC6019943

[11] S. Kageyama , S. R. Gudmundsson , Y. S. Sou , et al., “p62/SQSTM1‐Droplet Serves as a Platform for Autophagosome Formation and Anti‐Oxidative Stress Response,” Nature Communications 12, no. 1 (2021): 16. 10.1038/s41467-020-20185-1 PMC7782522 33397898

[12] V. Sood , K. B. Sharma , V. Gupta , et al., “ATF3 Negatively Regulates Cellular Antiviral Signaling and Autophagy in the Absence of Type I Interferons,” Scientific Reports 7, no. 1 (2017): 8789. 28821775 10.1038/s41598-017-08584-9 PMC5562757

[13] Y. Liu , J. Tang , D. Liu , et al., “Increased Autophagy in EOC Re‐Ascites Cells Can Inhibit Cell Death and Promote Drug Resistance,” Cell Death & Disease 9, no. 4 (2018): 419. 29549251 10.1038/s41419-018-0449-5 PMC5856849

[14] T. W. Kim , C. Cheon , and S. G. Ko , “SH003 Activates Autophagic Cell Death by Activating ATF4 and Inhibiting G9a Under Hypoxia in Gastric Cancer Cells,” Cell Death & Disease 11, no. 8 (2020): 717. 32879309 10.1038/s41419-020-02924-w PMC7468158

[15] J. Lu , W. Dong , H. He , et al., “Autophagy Induced by Overexpression of DCTPP1 Promotes Tumor Progression and Predicts Poor Clinical Outcome in Prostate Cancer,” International Journal of Biological Macromolecules 118 (2018): 599–609. 29874556 10.1016/j.ijbiomac.2018.06.005

[16] X. Qin , M. Liu , and X. Wang , “New Insights Into the Androgen Biotransformation in Prostate Cancer: A Regulatory Network Among Androgen, Androgen Receptors and UGTs,” Pharmacological Research 106 (2016): 114–122. 26926093 10.1016/j.phrs.2016.02.021

[17] W. Liu , X. Wang , Z. Liu , et al., “SGK1 Inhibition Induces Autophagy‐Dependent Apoptosis Via the mTOR‐Foxo3a Pathway,” British Journal of Cancer 117, no. 8 (2017): 1139–1153. 29017179 10.1038/bjc.2017.293 PMC5674106

[18] Z. Li , P. Chen , J. Chen , et al., “Glucose and Insulin‐Related Traits, Type 2 Diabetes and Risk of Schizophrenia: A Mendelian Randomization Study,” eBioMedicine 34 (2018): 182–188. 30100396 10.1016/j.ebiom.2018.07.037 PMC6116472

[19] J. Mateo , G. Seed , C. Bertan , et al., “Genomics of Lethal Prostate Cancer at Diagnosis and Castration Resistance,” Journal of Clinical Investigation 130, no. 4 (2020): 1743–1751. 31874108 10.1172/JCI132031 PMC7108902

[20] S. ChallaSivaKanaka , R. E. Vickman , M. Kakarla , S. W. Hayward , and O. E. Franco , “Fibroblast Heterogeneity in Prostate Carcinogenesis,” Cancer Letters 525 (2022): 76–83. 34715252 10.1016/j.canlet.2021.10.028 PMC8788937

[21] H. Liu , Q. Yang , Y. Xiong , Z. Xiong , and X. Li , “Improved Prognostic Prediction of Glioblastoma Using a PAS Detected From Single‐Cell RNA‐Seq,” Journal of Cancer 11, no. 13 (2020): 3751–3761. 32328180 10.7150/jca.44034 PMC7171486

[22] A. R. Lee , Y. Gan , Y. Tang , and X. Dong , “A Novel Mechanism of SRRM4 in Promoting Neuroendocrine Prostate Cancer Development Via a Pluripotency Gene Network,” eBioMedicine 35 (2018): 167–177. 30100395 10.1016/j.ebiom.2018.08.011 PMC6154886

[23] M. Bauckneht , C. Marini , V. Cossu , et al., “Gene's Expression Underpinning the Divergent Predictive Value of [18F]F‐Fluorodeoxyglucose and Prostate‐Specific Membrane Antigen Positron Emission Tomography in Primary Prostate Cancer: A Bioinformatic and Experimental Study,” Journal of Translational Medicine 21, no. 1 (2023): 3. 36600265 10.1186/s12967-022-03846-1 PMC9811737

[24] C. Yan and D. D. Boyd , “ATF3 Regulates the Stability of p53: A Link to Cancer,” Cell Cycle 5, no. 9 (2006): 926–929. 16628010 10.4161/cc.5.9.2714

[25] X. Yang , D. D. Yu , F. Yan , et al., “The Role of Autophagy Induced by Tumor Microenvironment in Different Cells and Stages of Cancer,” Cell & Bioscience 5 (2015): 14. 25844158 10.1186/s13578-015-0005-2 PMC4384293

[26] Y. Yu , Y. Song , L. Cheng , et al., “CircCEMIP Promotes Anoikis‐Resistance by Enhancing Protective Autophagy in Prostate Cancer Cells,” Journal of Experimental & Clinical Cancer Research 41, no. 1 (2022): 188. 35655258 10.1186/s13046-022-02381-7 PMC9161511

[27] J. Tosado , L. Zdilar , H. Elhalawani , et al., “Clustering of Largely Right‐Censored Oropharyngeal Head and Neck Cancer Patients for Discriminative Groupings to Improve Outcome Prediction,” Scientific Reports 10, no. 1 (2020): 3811. 32123193 10.1038/s41598-020-60140-0 PMC7051972

[28] M. Alawad , S. Gao , J. X. Qiu , et al., “Automatic Extraction of Cancer Registry Reportable Information From Free‐Text Pathology Reports Using Multitask Convolutional Neural Networks,” Journal of the American Medical Informatics Association 27, no. 1 (2020): 89–98. 31710668 10.1093/jamia/ocz153 PMC7489089

[29] K. N. Maxwell , H. H. Cheng , J. Powers , et al., “Inherited TP53 Variants and Risk of Prostate Cancer,” European Urology 81, no. 3 (2022): 243–250. 34863587 10.1016/j.eururo.2021.10.036 PMC8891030

[30] J. Dai , C. L. Hall , J. Escara‐Wilke , A. Mizokami , J. M. Keller , and E. T. Keller , “Prostate Cancer Induces Bone Metastasis Through Wnt‐Induced Bone Morphogenetic Protein‐Dependent and Independent Mechanisms,” Cancer Research 68, no. 14 (2008): 5785–5794. 18632632 10.1158/0008-5472.CAN-07-6541 PMC4432935

[31] M. Yuan , Y. Gao , L. Li , et al., “Phospholipase C (PLC)ε Promotes Androgen Receptor Antagonist Resistance Via the Bone Morphogenetic Protein (BMP)‐6/SMAD Axis in a Castration‐Resistant Prostate Cancer Cell Line,” Medical Science Monitor 25 (2019): 4438–4449. 31201297 10.12659/MSM.915828 PMC6590100

[32] T. C. Wei , H. Y. Lin , C. C. Lu , C. M. Chen , and L. R. You , “Expression of Crip2, a LIM‐Domain‐Only Protein, in the Mouse Cardiovascular System Under Physiological and Pathological Conditions,” Gene Expression Patterns 11, no. 7 (2011): 384–394. 21601656 10.1016/j.gep.2011.05.001

[33] L. Chen , N. Li , M. Zhang , et al., “APEX2‐Based Proximity Labeling of Atox1 Identifies CRIP2 as a Nuclear Copper‐Binding Protein That Regulates Autophagy Activation,” Angewandte Chemie 60, no. 48 (2021): 25346–25355. 34550632 10.1002/anie.202108961

[34] W. Shi , J. Bruce , M. Lee , et al., “MiR‐449a Promotes Breast Cancer Progression by Targeting CRIP2,” Oncotarget 7, no. 14 (2016): 18906–18918. 26934316 10.18632/oncotarget.7753 PMC4951339

